# Do all infants need vitamin D supplementation?

**DOI:** 10.1371/journal.pone.0195368

**Published:** 2018-04-12

**Authors:** Ane Cristina Fayão Almeida, Francisco José Albuquerque de Paula, Jacqueline Pontes Monteiro, Carlos Alberto Nogueira-de-Almeida, Luiz Antonio Del Ciampo, Davi Casale Aragon, Ivan Savioli Ferraz

**Affiliations:** 1 Department of Pediatrics, Ribeirão Preto Medical School, University of São Paulo—USP, Ribeirão Preto, Brazil; 2 Department of Medicine, Federal University of São Carlos—UFSCAR, São Carlos, Brazil; University of Alabama at Birmingham, UNITED STATES

## Abstract

A high prevalence of vitamin D deficiency (VDD) in children has been observed worldwide, but there are few studies on the nutritional status of vitamin D (VD) in healthy infants. The main cause of deficiency in healthy children is breastfeeding without supplementation and lack or insufficiency of sun exposure. The aims of this study were to determine serum concentrations of 25(OH)D and verify its association with parathyroid hormone (PTH) concentrations and use of VD supplementation in healthy infants aged ≥ 6 to ≤ 24 months attended at two Primary Health Care Units in Ribeirão Preto city, São Paulo, Brazil. A cross-sectional, observational and analytical study was performed in which serum concentrations of 25(OH)D, PTH, alkaline phosphatase (AP), calcium (Ca), phosphorus (P) and albumin were determined in 155 healthy infants. Information on sun exposure, sociodemographic aspects of mothers and clinical and nutritional characteristics of infants were obtained through interviews with responsible infants’s legal representatives. Ten infants (6%) presented deficient 25(OH)D serum concentration (≤20ng/ml) and 46 (30%), insufficient (21 to 29ng/ml). No changes in serum P, Ca and albumin concentrations were detected. Only one infant had an increase in PTH serum concentrations. 35% (55/155) of infants had high AP e 40% (22/55) presented insufficient serum concentrations of 25(OH)D but none presented deficient ones. There was a weak association between serum concentrations of 25(OH)D and PTH and an association between serum concentrations of 25(OH)D and P when adjusted for sex, age and BMI. There were no associations between inadequate serum concentrations of 25(OH)D (deficient ou insufficient), sun exposure and VD supplementation. This study found a low prevalence of deficient 25(OH)D serum concentration and high prevalence of insufficient ones which was not associated with changes in serum PTH, AP, P, Ca and albumin concentrations, VD supplementation and the formula volume intake.

## Introduction

Vitamin D (VD) is one of the fat-soluble vitamins. Approximately 90% of its requirement comes from sunlight exposure and its remaining from diet and/or dietary supplements[1]. In addition to having an important role in calcium homeostasis, vitamin D has several other functions in the body, such as inhibition of cell proliferation, stimulation of differentiation of cells of different strains, modulation of the immune system and inflammation [2].

The metabolic pathway for vitamin D synthesis begins with photochemical transformation of 7-dehydrocholesterol in several secosteroid compounds, including cholecalciferol (vitamin D3 –D3); this molecule undergoes two sequential hydroxylations for the production of the active metabolizing molecule 1,25(OH)_2_D. Vitamin D exerts its effects after binding with its receptor (vitamin D receptor—VDR). This, after binding with 1,25(OH)_2_D, forms heterodimers with retinoids X receptor (RXR) to exert its action by binding this complex to specific DNA sequences (vitamin D responsive elements) [2, 3].

Until recently, it was believed that the multiple actions of vitamin D in the body were produced only by the molecule 1,25(OH)_2_D. However, recent studies have shown that D3 can be converted to other secosteroids by the action of the enzyme cytochrome P450scc (CYP11A1). These secosteroids, especially 20(OH)D, 22(OH)D, 17(OH)D, 20,23(OH)_2_D, 20,22(OH)_2_D and 17,20,23(OH)_3_D were identified, among other sites, on the skin, adrenal, placenta and human serum. In human serum, the concentrations of 20(OH)D and 22(OH)D are 30 to 15 times lower than those of 25(OH)D, but greater than 1,25(OH)_2_D. Many of the molecules produced by the action of CYP11A1 may undergo further hydroxylations by other enzymes (CYP27B1, CYP24A1 and CYP27A1), producing new secosteroid compounds. These compounds appear to exert their functions as biased agonists when bound to the VDR or even as reverse agonists when combining with retinoic orphan acid receptors (ROR) α and γ. Many of these new secosteroids are biologically active, having reduced calcemic activity and may help explain the pleiotropic effects of vitamin D, especially with regard to anti-proliferative, pro-differentiation, and anti-inflammatory effects [2, 4–6].

Epidemiological studies have shown that vitamin D deficiency (VDD) is highly prevalente among infants in several countries, regardless of age, ethnicity, geographical location and climatic conditions and is believed to range from 2.7% to 45% [7–10].

Studies have shown that, as there is an increase in serum concentrations of 25(OH)D, a decrease in the proportions of elevated PTH concentrations is observed. However, to date no concentration of 25(OH)D has been found from which PTH values reach a plateau or are maximilly suppressed. Thus, even individuals with serum concentrations of 25(OH)D in values considered to be deficient (10 ng/ml) may present normal PTH values. In addition to calcemia, the relationship between serum concentrations of 25(OH)D and PTH may be affected by age; in identical serum concentrations of 25(OH)D, elevated serum PTH values are observed more frequently in elderly than in younger individuals [11].

In childhood, an additional variable is growth, usually marked by a positive balance of calcium, a mineral involved in bone mineralization, as well as its transportation that is facilitated by albumin, a major plasma protein [12, 13]. Most studies are not dedicated to the evaluation of 25(OH)D and parameters of mineral metabolism in the first two years of life [12, 14].

The main causes of inadequate serum 25(OH)D concentrations among healthy children are breastfeeding without supplementation and lack or insufficiency of sun exposure [1, 12, 15]. Exclusive breastfeeding provides from 11 to 38 IU/day [12, 15]. Exclusively breastfed infants present high risk of presenting inadequate serum 25(OH)D concentrations due to the decrease of 25(OH)D supply to breast milk in the eighth week of life [12].

Infants, defined by the World Health Organization [16] as a period between 0 and 24 months are more vulnerable to present inadequate serum 25(OH)D concentrations because of less sun exposure and long indoor period. There are few studies regarding serum concentrations of 25(OH)D in healthy infants. Therefore, the aim of this study was to determine serum concentrations of 25(OH)D and to verify its association with serum PTH concentrations and the use of VD supplementation in healthy infants aged ≥ 6 months and ≤ 24 months attended at two Primary Health Care Units of the Ribeirão Preto city, São Paulo, Brazil.

## Subjects and methods

### Design and subjects

It is a cross-sectional, observational and analytical study conducted in Ribeirão Preto city, São Paulo, which is located in the southeastern region of Brazil, at latitude 21°. It has a tropical climate, an means annual temperature of 23.2° C and 184 sunny days annually [17].

Two hundred and forty-five healthy infants, aged between 6 and 24 months, followed at two near municipal Primary Health Care Units, were invited to participate.

Six infants who were taking anticonvulsants and 14 infants on corticosteroids were not included in the study. Ninety infants were not included because their parents or responsible refused to participate. Thus, the sample consisted of 155 infants, 49 of one unit and 106 of another.

All mothers or guardians signed the consent. Data were collected within 12 months.

The study was approved by the Research Ethics Committee of the Center for Health School of the Medical School of Ribeirão Preto (Process HCRP No. 907.547), University of São Paulo (FMRP-USP).

### Procedures for data collection

After the agreement to participate in the study, 3ml of blood were collected from each infant, after 8-hour fasting, to analyze serum concentrations of 25(OH)D, PTH, alkaline phosphatase (AP), calcium (Ca), phosphorus (P) and albumin. The collected blood was transported in thermal box with recyclable ice, under temperature between 2º and 10ºC, and sent to the respective laboratories. After centrifugation, 600 μl of serum was stored at -80°C until the end of data collection for analysis of 25(OH)D and PTH in the laboratory of Endocrinology and Metabolism of the FMRP-USP and 600 to 800μl of serum was separated for analysis of Ca, P, AP and albumin that were determined every day in an automatic analyzer in the Biochemical laboratory of the FMRP. The intra and inter-assay errors were, respectively, less than 10 and 20% for the two parameters.

At the beginning of the study, a sociodemographic questionnaire was answered by mothers or guardians, and included questions such as the mother's age, educational level and family income. Infant clinical and nutritional was also obtained and included variables such as sex, age, skin color (classified according to the Brazilian Institute of Geography and Statistics [18] and observed by researcher), birth weight and breastfeeding. Questions about the hour, time and frequency of sun exposure, type of clothing when they exposed to the sun, cap or hat usage and the use of sunblock in the last seven days, were collected through a structured questionnaire elaborated by the researchers.

It was considered an adequate sun exposure if the infants had at least two of the following characteristics: sun exposure hour (10h to 15h), sun exposure time (> 15h), sun exposure frequency (> 3x/week), without wearing sunblock and type of clothing (short and blouse or body or diaper only, or naked). These criteria were adopted according to Specker and cols recommendations [19].

The VD intake of the infants was evaluated through questions answered by the mother or responsible, regarding the use of VD supplement in drops or infant formulas. In Brazil, infant formulas follow the Codex Alimentarius standard [20] and therefore contain 40UI to 100UI of VD for each 100 calories. Adequate supplementation of VD was considered when the infant used daily supplements (drops) containing at least 400 IU daily and/or ingested infant formula with volume ≥ 1 liter per day [12].

On the same day of blood collection, the infants were weighed on a digital infant device and measured with the stadiometer, according to the protocol of the Food and Nutrition Surveillance System [21]. The nutritional status was classified according to the growth curves of the World Health Organization [21].

### Biochemical analysis

The serum concentrations of 25(OH)D and PTH were analyzed by the chemiluminescence method (Diasorin® kit, Liaison®-Saluggia/Italy-25OH vitamin assay 310600 for 25(OH)D and Immulite®-Erlangen/Germany- for PTH). According to the criteria of the Endocrine Society Clinical Practice Guideline [22] deficient 25(OH)D serum concentration were considered when infants had values lower than ≤20ng/ml; values between 21 and 29ng/ml were considered insufficient and values above 30ng/ml were considered sufficient.

Serum PTH concentrations between 10 and 69 pg/ml were considered adequate [23]. The kinetic method optimized at 405 nm was used to determine AP [24]. The colorimetric method was applied for the determination of serum concentrations of albumin, Ca and P [25–27]. For the analyzes of AP, P, Ca and albumin the CT 600i equipment (Wiener Lab®-Rosario/Argentina) was used. The following reference values were considered: Ca (8.5 to 10.5 mg/dl), P (4.0 to 7.0 mg/dl), AP (250 to 645 U/L), albumin (3.5 to 4.8 g/dl) [24–27].

### Statistical analysis

The categorical variables (sociodemographic characteristics of the mother and clinical-nutritional characteristics of the infants and sun exposure) were set out in frequency tables. Serum concentrations of 25(OH)D, PTH, AP, Ca, P and albumin were expressed as mean and standard deviation. Contingency tables were elaborated to determine the association between categorical variables and nutritional status of 25(OH)D and the Fisher exact test was applied. Pearson’s correlation was used to determine the correlations between serum concentrations of 25(OH)D and PTH, AP, Ca, P and albumin; the Spearman's correlation was used to determine the correlations between serum concentrations of 25(OH)D and body mass Index (BMI) (z-score) and 25(OH)D serum concentrations and the age of infants.

Student's t-test was used to compare the mean serum concentrations of 25(OH)D between two groups regarding VD supplementation, duration of breastfeeding, use of sunblock and, time and duration of sun exposure.

ANOVA was used to compare mean serum concentrations of 25(OH)D with respect to sex, nutritional status, family income, seasons of the year, age, serum phosphorus concentrations and birth weight, as well as the mean serum P concentrations at different ages. In these cases, when the null hypothesis (equality between means) was rejected, Tukey's post-test was applied. ANCOVA was used to compare the mean serum concentrations of 25(OH)D regarding to the infant formula intake and use of VD supplementation (drops) with age adjustment.

Simple linear regression analysis was used to determine the association between serum concentrations of 25(OH)D, PTH, Ca, P, AP and albumin and multiple linear regression was used to compare these data adjusted for sex, age and BMI.

Prevalence ratios were determined to compare the prevalence of inadequate (deficient and insufficient) serum concentrations of 25(OH)D according to sun exposure and the use of VD supplementation by infants.

SAS software version 9.3 was used [28] for all statistical analyzes and significance level was set when value was below 5%.

## Results

### Characteristics of subjects

The majority of mothers had more than 20 years old, complete or higher elementary education and a monthly family income of less than or equal to 3 minimum wages. As for infants, the majority were males aged 6 to 11 months, white, had normal nutritional status and did not use infant formula. Among those who ingested formula, a large part consumed a volume between 500 and 1.000ml per day.

Table 1 shows the socio-demographic characteristics of mothers and clinical-nutritional characteristics of infants.

**Table 1:** Sociodemographic characteristics of the mothers and clinical-nutritional characteristics of healthy infants (n = 155) attended at two Primary Health Care Units of Ribeirão Preto, SP, Brazil, 2014–2015.

Variables	n (%)
*Sociodemographic characteristics of the mother*	
Age	
≤20 years old	13 (8)
>20 years old	142 (92)
Schooling	
Incomplete elementary school	36 (23)
Complete elementary school or higher	119 (77)
Monthly family income	
≤ 3 minimum wages	119 (77)
> 3 minimum wages	36 (23)
Profession	
Housewife	77 (50)
Working outside the home	78 (50)
*Clinical-nutritional characteristics of the infant*	
Gender	
Male	87 (56)
Female	68 (44)
Age	
6 to 11 months old	91 (59)
12 to 17 months old	27 (17)
18 to 24 months old	37 (24)
Skin color	
White	106 (68)
Brown	47 (30)
Black	2 (1)
Nutritional status	
Normal	122 (79)
Overweight and obesity	31 (20)
Underweigh	2 (1)
The infant is currently being breastfed	
Yes	73 (47)
Infant formula intake	
Yes	72 (46)
Volume of the infant formula	
< 1 liter	59 (38)
≥ 1 liter	13 (8)
Vitamin D supplements (drops)	
Yes	45 (29)

### Sun exposure

Most infants were exposed to the sun before 10:00 AM or after 3:00 PM, for more than 15 minutes, often less than three times a week and wore clothing that left legs and arms exposed.

Data related to the infant's sun exposure are shown in Table 2.

**Table 2:** Sun exposure of healthy infants (n = 155) attended at two Primary Health Care Units in Ribeirão Preto, São Paulo, Brazil, 2014–2015.

Variables	n (%)
*Sun exposure time*	
Before 10 a.m. and/or after 03 p.m.	144 (93)
From 10:00 a.m. to 03 p.m.	11 (7)
*Duration of sun exposure*	
≤ 15 min	66 (42)
> 15 min	89 (57)
*Frequency of sun exposure*	
≤ 3x/week	82 (53)
> 3x/week	73 (47)
*Type of clothing worn during sun exposure*	
Covering legs and arms	16 (10)
Head, legs and arms exposed	100 (64)
Only a diaper	34 (22)
Naked	5 (3)
*Use of a cap or hat*	
Yes	49 (32)
*Use of a sunscreen*	
Yes	31 (20)

### Biochemical profile

Ten (6%; 95% confidence interval– 95%CI:3.5 to 11.4) infants had serum concentrations of 25(OH)D considered deficient and 46 (30%; 95%CI: 16.4 to 42.8) insuficient. Ninety-nine infants (64%; 95% confidence interval–CI: 54.4 to 73.3) had adequate serum concentrations of 25(OH)D.

Serum concentrations of Ca, P and albumin were under normal range; only one male infant had elevated serum PTH levels, and he presented with insufficient 25(OH)D concentration (23.5ng/ml); 35% (55/155) of the infants had high AP, and 56% (31/55) of them were under one year of age; among infants with elevated serum concentrations of AP, 93% (51/55) were had normal nutritional status and none presented deficient serum concentrations of 25(OH)D but 40% (22/55) presented insufficiency.

Table 3 shows mean and standard deviations of serum concentrations of 25(OH)D, PTH, Ca, P, AP and albumin of the evaluated infants.

**Table 3:** Serum concentrations of calcidiol [25(OH)D], parathormne, calcium, phosphorus, alkaline phosphatase and albumin of healthy infants attended at two Primary Health Care Units in Ribeirao Preto, São Paulo, Brazil, 2014–2015.

Variable	Mean (SD)
25(OH)D (ng/ml)	34 (10)
PTH (pg/ml)	16 (11)
Ca (mg/dl)	10.5 (0.5)
P (mg/dl)	6 (1)
AP (U/L)	616 (250)
Albumin (g/dl)	4.4 (0.2)

PTH = parathormone; P = phosphorus

Ca = calcium; AP = alkaline phosphatase

### 25(OH)D and PTH

**Fig 1:**
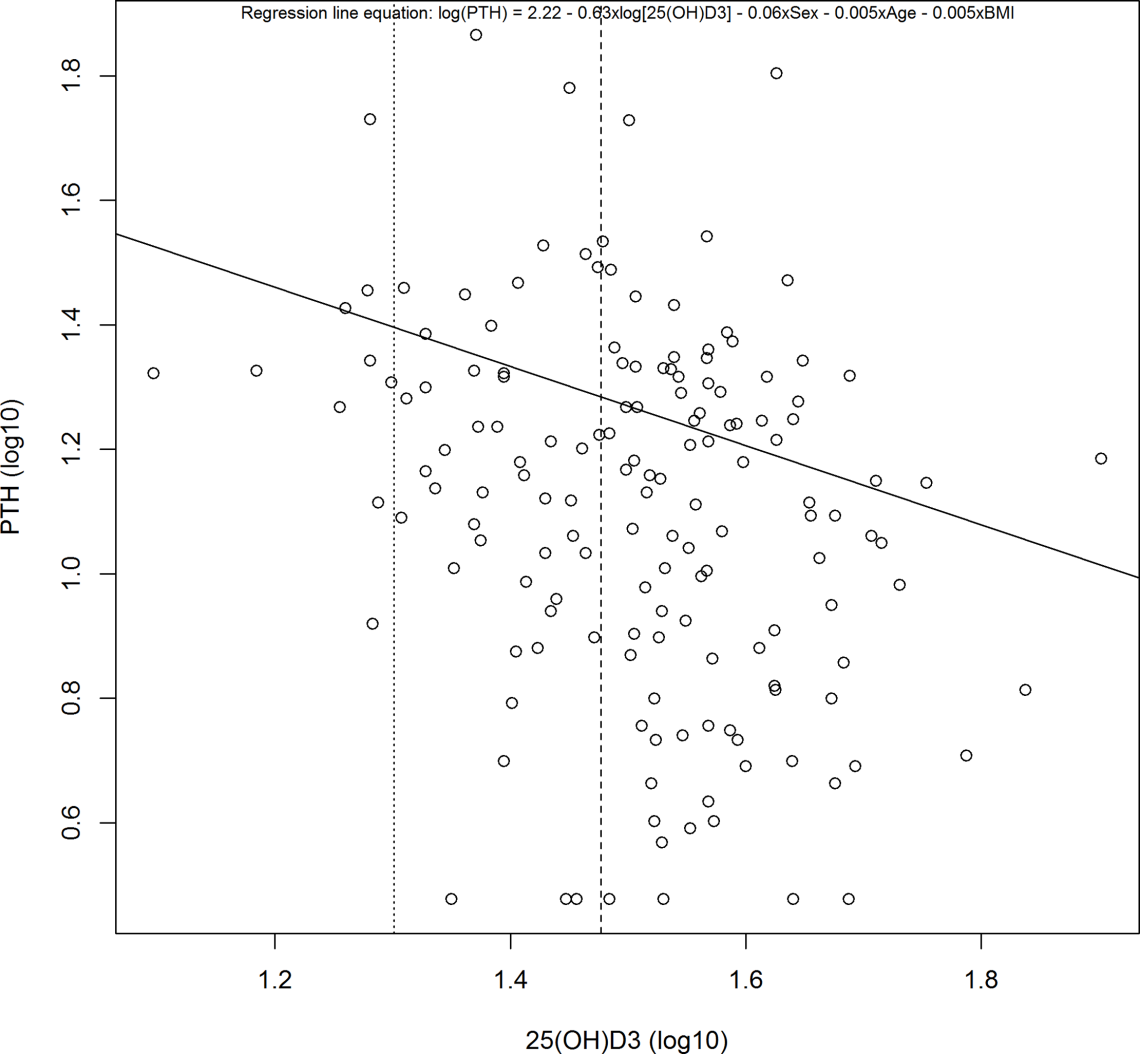
Linear association between serum concentrations of 25(OH)D and PTH in healthy infants attended at two Primary Health Care Units in Ribeirao Preto, São Paulo, Brazil, 2014–2015.

There was a weak inverse correlation between serum concentrations of log_10_ 25(OH)D and log_10_ PTH in the infants [parameter estimate (95%CI) = -0.63 (-0.99; -0.28); p-value < 0.01)], even after adjustment for covariates (sex, age and BMI). However, a plateau of serum PTH levels was not observed when concentrations of 25(OH)D reached 20ng/ml or 30ng/ml, as shown in Fig 1.

### Correlations between serum 25(OH)D concentrations and Ca, P, AP, albumin, BMI and age

There was no correlation between serum concentrations of 25(OH)D and Ca, P, AP and albumin. There were also no correlations between serum concentrations of 25(OH)D and z-scores of BMI (r = 0.10, p-value = 0.23). Furthermore, no correlation was found between the age of the infants and the serum concentrations of 25(OH)D (r = -0.07; p-value = 0.37).

There were no differences in 25(OH)D serum levels regarding season of the year, age (6–11 months, 12–17 months and 18–24 months), sex of the infant, monthly family income, birth weight and nutritional status, as shown in Table 4. Regarding the serum concentrations of phosphorus in the different age groups, it was observed that infants less than 1 year old had serum concentrations higher than the other age groups.

**Table 4:** Comparison of mean serum 25(OH)D concentrations of healthy infants attended at two Primary Health Care Units according to seasons, age, sex, monthly family income, birth weight and nutritional status (Ribeirão Preto, São Paulo, Brazil, 2014–2015).

Variables	n	Mean (SD)	p-value
*Seasons*			
Spring	48	34 (11)	
Summer	37	37 (9)	0.07
Autumn	36	33 (8)	
Winter	34	31 (11)	
*Age*			
6m to 11m	91	35 (11)	
12m to 17m	27	31 (9)	0.18
18m to 24m	37	33 (7)	
*Sex*			
Male	87	34 (10)	0.62
Female	68	33 (10)	
*Family income*			
< 3 minimun wage	119	34 (10)	0.76
≥ 3 minimun wage	36	34 (10)	
*Birth weight*			
Insuficient weight	48	33 (7)	
Adequate	81	33 (11)	0.53
Weight excess	26	36 (12)	
*Nutritional Status*			
Normal weight	122	34 (10)	0.42
Overweight + obesity	11	31 (8)	

No differences were found between the means of 25(OH)D serum concentrations among infants according to sex, family income, use of VD supplementation (drops), use of sunscreen, time and duration of sun exposure, nutritional status and breastfeeding (Table 5).

**Table 5:** Comparison of mean serum 25(OH)D concentrations of healthy infants attended at two Primary Health Care Units according to VD supplementation (drops), infant formula intake, characteristic of sun exposure and breastfeeding (Ribeirão Preto, São Paulo, Brazil, 2014–2015).

Variable	Category	N	Mean (SD)	p-value
Use of VD supplementation (drops)	Yes	45	35 (13)	0.29
	No	110	33 (9)	
Formula volume	<1000 ml/day	142	33 (10)	0.09
	≥1000 ml/day	13	40 (14)	
Use of a sunblock	Yes	31	33 (9)	0.49
	No	124	34 (10)	
Nutritional status	Normal weight	122	34 (10)	0.42
	Overweight + obese	31	31 (8)	
Duration of solar exposure	≤ 15 min	66	34 (11)	0.96
	> 15 min	89	34 (10)	
Time of solar exposure	Before 10 a.m. and/or after 03 p.m.	144	34 (10)	0.35
	From 10 a.m. to 03 p.m	11	31 (9)	
Breastfeeding	Yes	73	34 (12)	0.64
	No	82	33 (9)	

#### Comparison between infant formula intake and serum concentrations of 25(OH)D

We did not found any association between VD suplementation (VD supplementation in drop and/or infant formula ≥ 1 liter) and inadequate (deficient or insufficient) serum concentrations of 25(OH)D (Fisher's exact test, p-value = 0.06).

**Fig 2:**
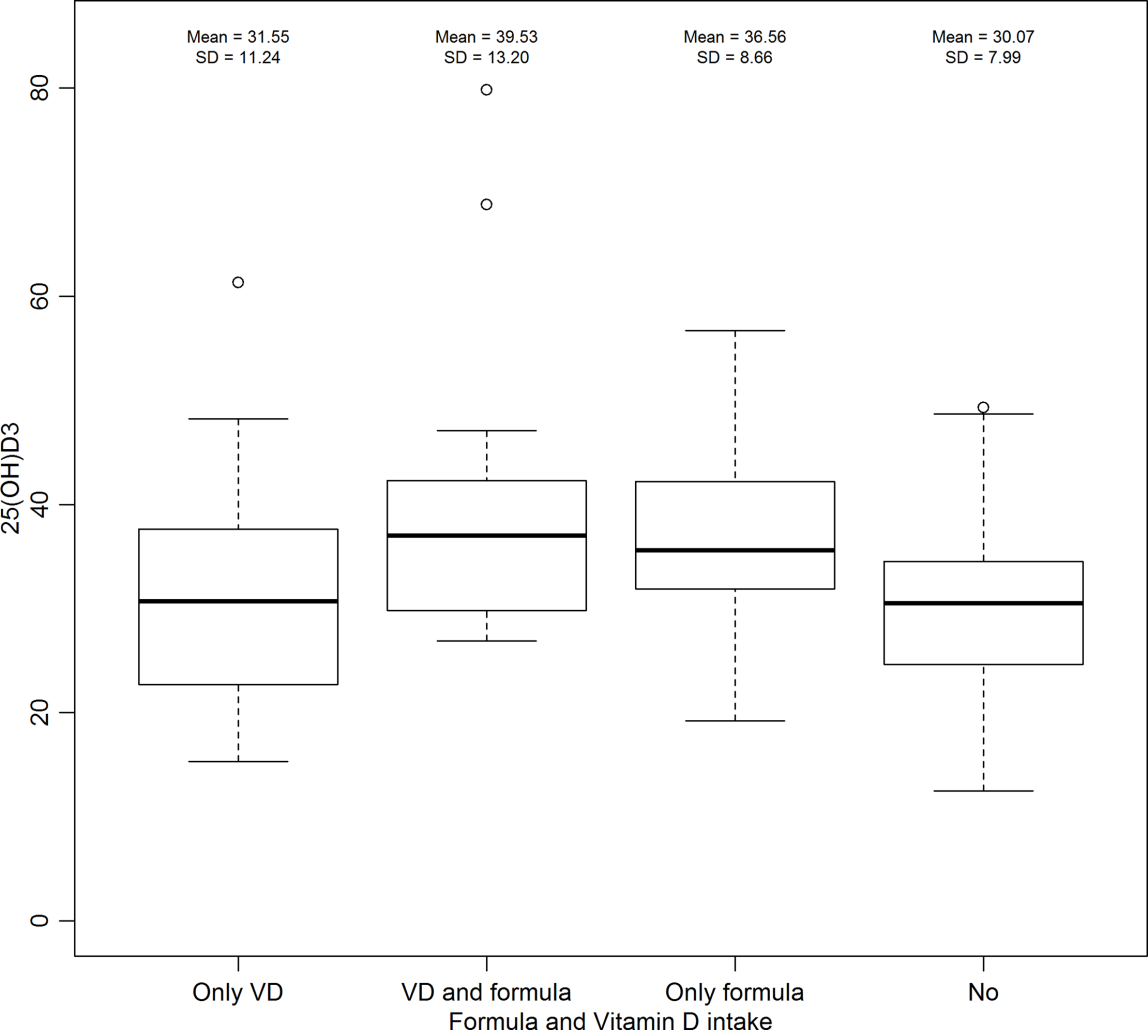
Comparison between consumption of infant formula and VD supplementation and serum concentrations of 25(OH)D in healthy infants attended at two Primary Health Care Units (Ribeirão Preto, São Paulo, Brazil, 2014–2015).

Fig 2 shows that infants who did not drink infant formula, even using VD supplementation, had significantly lower serum concentrations of 25(OH)D than those who had used infant formula.

As observed, there was difference in the mean serum concentrations of 25(OH)D among infants who consumed infant formula in relation to those who did not use it; infants who consumed infant formula had higher concentrations of 25(OH)D even compared to those who used VD supplementation but had not ingested formula. When the ANCOVA was adjusted for age, the p-value for formula use was <0.01 showing differences among class 1 (children who ingested only VD supplementation) and 4 (infant who did not ingest formula or VD supplementation) in relation to 2 (children who ingested infant formula and VD supplementation) and 3 (children who just used infant formula). However, mean serum concentrations of 25(OH)D in all ranges of infant formula intake were within the normal values.

There was no association between serum concentrations of 25(OH)D and Ca, AP and albumin. The same pattern was maintained with the model adjusted for sex, age and BMI. It was observed an association between 25(OH)D and PTH concentrations in the simple model (parameter estimate (95%CI) = -0.61 (-0.96; -0.25); p-value < 0.01) and after adjustment for sex, age and BMI in the multiple model (parameter estimate (95%CI) = -0.63 (-0.99; -0.28); p-value < 0.01). In the multiple model, it was observed an association between serum P concentrations with 25(OH)D when adjusted for sex, age and BMI (p-value = 0.04, r^2^ = 0.25). This finding did not occur in the simple model (p-value = 0.18; r^2^ = 0.01). Infants aged 6 to 11 months had serum concentrations of P greater than infants over 12 months old (ANOVA, p-value <0.01).

Table 6 shows the prevalence ratios and their 95% CIs among different groups of infants according to adequacy and inadequacy of sun exposure and VD supplementation. There was no association between the prevalence of deficient or insufficient serum concentrations of 25(OH)D, sun exposure and vitamin supplementation.

**Table 6:** Frequency and prevalence ratios of deficiency, insufficiency and sufficiency serum concentration of 25(OH)D in healthy infants attended at two Primary Health Care Units according to the adequacy of sun exposure and VD supplementation (Ribeirão Preto, São Paulo, Brazil, 2014–2015).

	25(OH)D Deficiency (≤20ng/ml)	25(OH)D Insufficiency (21 – 29ng/ml)	25(OH)D Sufficiency (≥30ng/ml)	Total	Prevalence ratios (95% CI****) for deficiency	Prevalence ratios (95% CI) for insuficiency
Adequate sun exposure (at least 2 factors*) and adequate VD supplementation**	**3 (7%)**	**9 (23%)**	**27 (70%)**	**39**	**reference**	**reference**
**Adequate sun exposure (at least 2 factors) and inadequate VD supplementation**	**2 (3%)**	**27 (34%)**	**48 (63%)**	**77**	**0.34** **(0.06–1.94)**	**1,51 (0,79; 2,91)**
**Inadequate sun exposure (at least 2 factors) and adequate VD supplementation**	**0 (0)**	**5 (42%)**	**7 (58%)**	**12**	**-** ***	**1,80 (0,75; 4,36)**
**Inadequate sun exposure (at least 2 factors) and inadequate VD supplementation**	**5 (18%)**	**4 (15%)**	**18 (67%)**	**27**	**2.41** **(0.63–9.24)**	**0,64 (0,22; 1,87)**

Abbreviation: VD = vitamin D

*Sun exposure time from 10:00 a.m. to 3:00 p.m., duration of sun exposure >15 min, frequency of solar exposure at least 3 times a week, no use of a sunscreen, and type of clothing worn (shorts, shirt or jumper, or only a diaper, or naked).

**Intake of daily supplement drops containing at least 400 IU D3 and/or an intake ≥1 liter of infant formula per day.

*** The sampling zero did not permit a calculation of the prevalence ratio

****95%CI: 95% confidence interval

## Discussion

There are few studies [3,4,26,27,28] regarding nutritional status of the VD in healthy infants. In the present study, 6% of the infants presented deficient serum 25(OH)D concentrations and 30%, insufficient. The low prevalence of deficient serum 25(OH)D concentrations observed, especially when compared to the aforementioned studies, can probably be explained by the fact that the Ribeirão Preto city has a tropical climate, with good conditions of insolation; in addition, it should be taken into account that only healthy infants participated in the study; however, these conditions may not be sufficient to guarantee adequate serum 25(OH)D concentrations in a portion of the population, due to the high prevalence of insufficient values observed. Some factors—very difficult to assess in practice—adult dependence on sun exposure, lack or low compliance of VD supplementation, and low consumption of VD rich foods may help explain the high prevalence of insufficient concentrations of 25(OH)D observed in this population. High prevalences of VD insufficiency, using the cut-off points of the Endocrine Society [22], were observed in two other studies. Abdul-Razzak and cols study [29] reported a prevalence of 28.4% of insufficiency (<30ng/ml) and 11.3% of deficiency (<20ng/ml) in healthy Jordanian infants and Dyson and cols [30] reported a prevalence of 19.4% of VD insufficiency among Australian children.

There is no consensus in the literature regarding cut-off points to define serum concentrations of 25(OH)D as deficient, insufficiency and adequacy [12, 31–34]. Many studies have used the cut-off points of the Endocrine Society [7, 8, 22].However, some authors have defined VDD in infants and children as 25(OH)D serum concentrations lower than 20 ng/ml (50 nmol/l) and adequate when 25 (OH) D concentrations are greater than 20 ng/ml [29, 31, 35–41].

It was found that infants under one year had significantly higher mean serum concentrations of P than infants above this age group. It is known that in the first year of life, bone growth and mineralization are rapid [42], a fact that may justify the observation of higher values of P in the first year of life, although within the normal range. In relation to AP, 35.5% of the infants presented high values. Considering that more than half of the infants with elevated AP were less than one year old and that there was no correlation between serum concentrations of 25(OH)D and AP, a possible explanation for this finding in the present study is that it may be transient in childhood, a common condition found in healthy infants and may occur without the presence of bone or liver diseases and are not associated with the status of VD [43, 44].

There was no difference in the means of 25(OH)D concentrations in relation to birth weight. as well as Grant et al [9], in which there was no association between 25(OH)D concentrations and birth weight of infants. In the present study, the absence of infants born with low birth weight and the fact that all the birth weight groups present mean 25(OH)D serum concentrations within the values considered adequate may help to explain this finding. On the other hand, studies have shown that mothers with higher concentrations of 25(OH)D (eg> 40 ng/ml), especially in periods close to parturition, present a significant reduction in the rate of prematurity, especially when compared to those with concentrations <20ng / ml [45, 46].

In the present study, there were no associations between serum concentrations of 25(OH)D and Ca, AP and albumin, which can be explained by the low prevalence of values of 25(OH)D considered deficient. However, it was observed association between serum concentrations of 25(OH)D and PTH levels. Although such an association was observed, clinically it was not relevant because only one male infant presented with elevated serum PTH concentrations but he didn’t have 25(OH)D deficiency. There were also associations of 25(OH)D and P concentrations after adjustment for covariates. In this case, probably, the association was influenced by the serum concentrations of P, which were higher in infants less than one year. Few studies have found an association between concentrations of 25(OH)D and PTH, Ca, P and AP in children. In addition, in those studies a high prevalences of deficient serum concentrations of 25(OH)D and changes in PTH, Ca, AP and P are registred [8, 10, 47].

Sai and cols [48] have shown that plateau or PTH balance levels are reached when serum concentrations of 25(OH)D are close to 20 ng/ml or 30 ng/ml; these authors stands out that these would be the minimum values desired to obtain the benefits of VD in the various systems. Valcour and cols [11] observed a significant decline in PTH concentrations when 25(OH)D was between 20 and 30ng/ml. In the present study, the plateau of PTH serum concentrations wasn’t observed when serum concentrations of 25(OH)D reached values of 20 or 30ng/ml, there maybe other biological markers, in addition to VD, that interfere with PTH concentrations. Furthermore, we observed a weak inverse correlation between serum concentrations of 25(OH)D and PTH, a fact that can be explained by the high prevalence of insufficient serum concentrations of 25(OH)D, but with a low prevalence of values considered deficient. Carrol and cols [47] and Halicioglu and cols [10] reported an inverse correlation between serum concentrations of 25(OH)D and PTH in children and adolescents, but both studies observed high prevalences of deficient serum 25(OH)D concentrations.

In the current study, there was no association between insufficient or deficient serum concentrations of 25(OH)D, sun exposure and VD supplementation. Studies conducted in Ireland (latitude 53°) [47], New Zealand (latitude 40°) [9] and in the USA (latitude 33°) [8] didn’t show association between sun exposure and deficient serum 25 (OH) D concentrations. However, in some of these studies the authors didn’t take into account the time and frequency of sun exposure, sunblock use, type of clothing, and among other factors that influence VD synthesis [10, 29, 47, 49].

The present study failed to show an association between deficient serum 25(OH)D concentrations and use of drop supplementation. One of the explanations for this fact is that study was developed in a place with good conditions of sun exposure; in addition, it should be noted that, according to our criteria, more than 75% of infants had adequate sun exposure, therefore VD supplementation may play a secondary role in the prevention of vitamin D deficiency. Similar results were found by Akman and cols [50] that demonstrated an absence of association between deficient serum 25(OH)D concentrations and supplementation in the first year of life. On the other hand, in two European studies—Poland [51] and Ireland [47]- a positive correlation was found between VD supplementation, ranging from 400 IU/day to 4000 IU/day, and serum concentrations of 25(OH)D.

It was noted that children who consumed infant formula had higher serum concentrations of 25(OH)D than those who didn’t, even after adjusting for age; this difference was observed even when compared to infants who didn’t use infant formula but consumed VD supplementation. However, the mean of 25(OH)D was greater than 30ng/ml in all infants, regardless of the use of infant formulas. In a study conducted in Ireland [47], the authors observed that infants who drank infant formula had higher 25(OH)D serum concentrations than those who drank cow's milk (fortified or not). It was also observed that the use of infant formula during the first six months of life was associated with higher serum concentrations of 25(OH)D, even after adjustments for age, in an American study [8].

Although it is the most important "natural source" of VD synthesis, safe exposure limits are not known [52], Some studies have shown a relationship between sun exposure and the development of some types of skin cancer [53, 54]. Therefore the American Academy of Pediatrics[55] recommends that children who do not ingest 600 IU of vitamin D from diet or from a formula with volume greater than 1 liter per day, as well as children who do not have regular sun exposure, should receive prophylactic VD supplementation as a VDD prevention. On the other hand, the Institute of Medicinep[33] and the American Academy of Pediatrics[56] do not recommend regular sun exposure orientation for VD synthesis.

Considering the significant increase in the prescription of prophylactic VD supplementation, attention should be given to the overdose that may lead to toxicity. While prolonged sun exposure causes no intoxication [1], this may occur due to excessive supplementation of VD that, although rare, may cause clinical manifestations due to hypercalcemia and hypercalciuria, such as nausea, vomiting, abdominal pain, nephrolithiasis and central nervous system depression[57].

In temperate countries, some authors[35, 58] showed a higher prevalence of deficient serum 25(OH)D concentrations in infants evaluated in autumn and winter, coinciding with the period of less exposure to sunlight and lower synthesis efficiency during exposure. In the present study there were no diferences in the mean concentrations of 25(OH)D regarding the different season of the year.

Two Amercian studies [7, 58] showed that black children had a higher prevalence of deficient serum 25(OH)D concentration; in addition, another American study conducted in Hawaii [59] found that children younger than 5 years old with darker skin had, in general, higher rates of hospitalization secondary to bronchiolitis and pneumonia, a fact that could be explained by the lower serum concentrations of 25(OH)D commonly observed in these individuals [60, 61]. On the other hand, in an Arab study [36] researchers found no association between skin color and deficient serum 25(OH)D concentrations. In the present study, only two infants were black, a fact that didn’t allow this association; moreover, one had adequate serum 25(OH)D concentrations (47.4 ng/ml) and the other had insufficient serum concentrations (23 ng/ml).

There was no difference in serum concentrations of 25(OH)D regarding sex. Similar results were found in studies in Jordan, Turkey and Spain [10, 35, 61]. The absence of difference in sun exposure and the use of VD supplementation between the sex, together with the fact that most infants had adequate sun exposure, may explain these findings.

In the present study, no correlation was observed between age of infants and their respective serum concentrations of 25(OH)D. Carpenter and cols [8] and Alonso and cols [35] found an inverse correlation between age and 25(OH)D serum concentrations in American and Spanish infants, respectively. In some studies [8, 58, 62, 63] conducted with infants and children, researchers found that 25(OH)D serum concentrations decreased with age, with a higher proportion of deficient values found in children over two years of age.

It is known that in obesity there is a sequestration of part of the VD in adipose tissue and, in fact, some researchers have observed a relation between overweight and VDD in infants and children [36, 47, 64, 65]. However, Alonso and cols [35] didn’t find a correlation between serum concentrations of 25(OH)D and BMI in infants and Spanish children. In the present study, this association was also not observed.

The present study is the first study in Brazil to determine the serum concentrations of 25(OH)D along with the serum concentrations of PTH, Ca, P and AP, as well as to verify the relationship of this vitamin with sun exposure and the use of vitamin supplementation in healthy infants.

This study has some limitations. The study population was obtained from a convenience sample and can not be considered as representative of infants from Ribeirão Preto. In addition, in order to estimate the sun exposure, a questionnaire developed by authors was used since there are no validated questionnaires for this purpose. Althogh as a form of prevention of vitamin D deficiency some researchers recommend a sensible sun exposure, that is, an exposure that does not cause burns to the skin [66], it wasn’t possible to accurately estimate the percentage of the body surface of the infants exposed to the sun by the questionnaire. Therefore these estimates might be imprecise in view of the large number of factors that influence the personal ultraviolet B irradiation from sunlight and cutaneous VD synthesis [64]. Likewise, considering that the VD supplementation was not provided by the researchers, it was not possible to estimate compliance to the treatment and this may have influenced the results found. The same observation can be made regarding infant formulas, since they were also not provided by researchers or health units. In our study we didn’t perform the vitamin D dosage contained in infant formulas consumed by infants but, in Brazil, infant formulas follow the Codex Alimentarius standard [20] and therefore contain 40UI to 100UI of VD for each 100 calories. It should also be taken into account that information obtained through questionnaires may contain memory biases. It was decided not to use a food survey to evaluate the VD intake since the greater synthesis of the vitamin occurs through sun exposure. Finally, changes in the serum concentrations of some biochemical markers that occur late to changes in the values of other markers may not have been detected because they were dosed only once.

In conclusion, this study found a low prevalence of deficient serum 25(OH)D concentrations, but high values of insufficiency which wasn’t correlated with changes in serum PTH, AP, P, Ca and albumin concentrations, sun exposure and with VD supplementation. Due to the lack of safe limits of sun exposure for infants, it is recommended the prophylactic VD supplementation for infants, however, considering the side effects of intoxication of VD, the real need for the use of supplementation should be evaluated, especially in communities with good insolation condition.
